# Second and Secondary Lattice Modules

**DOI:** 10.1155/2014/291924

**Published:** 2014-10-14

**Authors:** Fethi Çallıalp, Ünsal Tekir, Emel Aslankarayiğit Uğurlu, Kürşat Hakan Oral

**Affiliations:** ^1^Department of Mathematics, Beykent University, Ayazaga-Maslak, 34396 Istanbul, Turkey; ^2^Department of Mathematics, Marmara University, Ziverbey, Göztepe, 34722 Istanbul, Turkey; ^3^Department of Mathematics, Yildiz Technical University, 34210 Istanbul, Turkey

## Abstract

Let *M* be a lattice module over the multiplicative lattice *L*. A nonzero *L*-lattice module *M* is called second if for each *a* ∈ *L*, *a*1_*M*_ = 1_*M*_ or *a*1_*M*_ = 0_*M*_. A nonzero *L-*lattice module *M* is called secondary if for each *a* ∈ *L*, *a*1_*M*_ = 1_*M*_ or *a* ^*n*^1_*M*_ = 0_*M*_ for some *n* > 0. Our objective is to investigative properties of second and secondary lattice modules.

A multiplicative lattice *L* is a complete lattice in which there is defined as a commutative, associative multiplication which distributes over arbitrary joins and has the compact greatest element 1_*L*_ (least element 0_*L*_) as a multiplicative identity (zero). An element *a* ∈ *L* is said to be proper if *a* < 1_*L*_. An element *p* < 1_*L*_ in *L* is said to be prime if *ab* ≤ *p* implies either *a* ≤ *p* or *b* ≤ *p*. If 0_*L*_ is prime, then *L* is said to be a domain. For *a* ∈ *L*, we define $\sqrt{a}=⋁\left\{x\in L:x^{n}\leq a\;\text{for}\;\text{some}\;\text{integer}\;n\right\}$. An element *p* < 1_*L*_ in *L* is said to be primary if *ab* ≤ *p* implies either *a* ≤ *p* or $b\leq \sqrt{p}$.

If *a*, *b* belong to *L*, (*a*:_*L*_ *b*) is the join of all *c* ∈ *L* such that *cb* ≤ *a*. An element *e* of *L* is called meet principal if *a*⋀*be* = ((*a*:_*L*_ *e*)⋀*b*)*e* for all *a*, *b* ∈ *L*. An element *e* of *L* is called join principal if ((*ae*⋁*b*):_*L*_ *e*) = *a*⋁(*b*:_*L*_ *e*) for all *a*, *b* ∈ *L*. *e* ∈ *L* is said to be principal if *e* is both meet principal and join principal. *e* ∈ *L* is said to be weak meet (join) principal if *a*⋀*e* = *e*(*a*:_*L*_ *e*)  (*a*⋁(0_*L*_:_*L*_ *e*) = (*ea*:_*L*_ *e*)) for all *a* ∈ *L*. An element *a* of a multiplicative lattice *L* is called compact if *a* ≤ ⋁*b*_*α*_ implies *a* ≤ *b*_*α*_1__⋁*b*_*α*_2__⋁⋯⋁*b*_*α*_*n*__ for some subsets {*α*_1_, *α*_2_,…, *α*_*n*_}. If each element of *L* is a join of principal (compact) elements of *L*, then *L* is called a *PG*-lattice (*CG*-lattice). If *L* is a *CG*-lattice and *p* is a primary element, then $\sqrt{p}$ is prime [1, Lemma 2.1].

Let *M* be a complete lattice. Recall that *M* is a lattice module over the multiplicative lattice *L* or simply an *L*-module in case there is a multiplication between elements of *L* and *M*, denoted by *lB* for *l* ∈ *L* and *B* ∈ *M*, which satisfies the following properties:

1. (*lb*)*B* = *l*(*bB*);
2. (⋁_*α*_ *l*_*α*_)(⋁_*β*_ *B*_*β*_) = ⋁_*α*,*β*_ *l*_*α*_ *B*_*β*_;
3. 1_*L*_ *B* = *B*;
4. 0_*L*_ *B* = 0_*M*_;

for all *l*, *l*_*α*_, *b* in *L* and for all *B*, *B*_*β*_ in *M*.

Let *M* be an *L*-module. If *N*, *K* belong to *M*, (*N*:_*L*_ *K*) is the join of all *a* ∈ *L* such that *aK* ≤ *N*. Particularly, (0_*M*_:_*L*_1_*M*_) is denoted by *ann*(*M*). If *a* ∈ *L* and *N* ∈ *M*, then (*N*:_*M*_ *a*) is the join of all *H* ∈ *M* such that *aH* ≤ *N*. An element *N* of *M* is called meet principal if (*b*∧(*B*:_*L*_ *N*))*N* = *bN*∧*B* for all *b* ∈ *L* and for all *B* ∈ *M*. An element *N* of *M* is called join principal if *b*∨(*B*:_*L*_ *N*) = ((*bN*∨*B*):_*L*_ *N*) for all *b* ∈ *L* and for all *B* ∈ *M*. *N* is said to be principal if it is both meet principal and join principal. In special cases, an element *N* of *M* is called weak meet principal (weak  join  principal) if (*B*:_*L*_ *N*)*N* = *B*∧*N*  ((*bN*:_*L*_ *N*) = *b*∨(0_*M*_:_*L*_ *N*)) for all *B* ∈ *M*  (for  all  *b* ∈ *L*). *N* is said to be weak principal if *N* is both weak meet principal and weak join principal.

Let *M* be an *L*-module. An element *N* in *M* is called compact if *N* ≤ ⋁_*α*_ *B*_*α*_ implies *N* ≤ *B*_*α*_1__∨*B*_*α*_2__∨⋯∨*B*_*α*_*n*__ for some subsets {*α*_1_, *α*_2_,…, *α*_*n*_}. The greatest element of *M* will be denoted by 1_*M*_. If each element of *M* is a join of principal (compact) elements of *M*, then *M* is called a *PG*-lattice module (*CG*-lattice module).

Let *M* be an *L*-module. An element *N* ∈ *M* is said to be proper if *N* < 1_*M*_. For all elements *N* of *M*, [*N*, 1_*M*_] is a set of all *K* ∈ *M* such that *N* ≤ *K* ≤ 1_*M*_ and [*N*, 1_*M*_] is an *L*-lattice module with *a* · *K* = *aK*∨*N* for all *a* ∈ *L* and *K* ∈ *M* such that *N* ≤ *K*.

For various characterizations of lattice modules, the reader is referred to [2–9].

**Definition 1 .**

A nonzero *L*-lattice module *M* is called second if for each *a* ∈ *L*, *a*1_*M*_ = 1_*M*_ or *a*1_*M*_ = 0_*M*_.

**Definition 2 .**

A nonzero *L*-lattice module *M* is called secondary if for each *a* ∈ *L*, *a*1_*M*_ = 1_*M*_ or *a* ^*n*^1_*M*_ = 0_*M*_ for some *n* > 0.

**Example 3 .**

Let *Z* be the integers, let *Q* be the rational numbers, and let *Q* be *Z*-module. Suppose *L* = *L*(*Z*) is the set of all ideals of *Z* and *M* = *L*(*Q*) is the set of all submodules of *Q*. Thus, *M* as *L*-lattice module is a second module, since for every integer *n* ∈ *Z*, (*nZ*)*Q* = *Q* or (*nZ*)*Q* = 0.

**Remark 4 .**

Every second lattice module is a secondary lattice. But the converse is not true. For this, we can give the following example.

**Example 5 .**

Let *Z* be the integers and let *Z*_4_ be *Z*-module. Suppose that *L* = *L*(*Z*) is the set of all ideals of *Z* and *M* = *L*(*Z*_4_) is the set of all submodules of *Z*_4_. Thus, *M* as *L*-lattice module is a secondary lattice module, which is not a second lattice module.

**Example 6 .**

Let *Z* be the integers and *L* = *L*(*Z*) the set of all ideals of *Z*. Thus, *L* as *L*-lattice module is neither a second lattice module nor a secondary lattice module.

**Proposition 7 .**

Let *L* be a *CG*-lattice and let *M* be a nonzero *L*-lattice module. If for each compact *a* ∈ *L*, *a*1_*M*_ = 1_*M*_ or *a*1_*M*_ = 0_*M*_, then *M* is a second *L*-lattice module.

**Proof**

Let *r* ∈ *L*. Since *L* is a *CG*-lattice, then we have *r* = ⋁_*i*_ *c*_*i*_ such that *c*_*i*_'s are compact elements of *L*. Then, we obtain *r*1_*M*_ = (⋁_*i*_ *c*_*i*_)1_*M*_ = ⋁_*i*_ *c*_*i*_1_*M*_. We have two cases.

*Case 1.* If *c*_*i*_1_*M*_ = 0_*M*_ for each compact *c*_*i*_ ∈ *L*, then we have *r*1_*M*_ = (⋁_*i*_ *c*_*i*_)1_*M*_ = ⋁_*i*_ *c*_*i*_1_*M*_ = 0_*M*_.

*Case 2.* If *c*_*i*_1_*M*_ = 1_*M*_ for some compact *c*_*i*_ ∈ *L*, then we have *r*1_*M*_ = (⋁_*i*_ *c*_*i*_)1_*M*_ = ⋁_*i*_ *c*_*i*_1_*M*_ = 1_*M*_.

Hence, *r*1_*M*_ = 1_*M*_ or *r*1_*M*_ = 0_*M*_ for each *r* ∈ *L*. Consequently, *M* is second.

**Proposition 8 .**

If *M* is a second *L*-lattice module, then *ann*(*M*) = (0_*M*_:_*L*_1_*M*_) = *p* is a prime element of *L*. In this case, *M* is called *p*-second lattice module.

**Proof**

Suppose that *M* is a second *L*-lattice module. Clearly, *ann*(*M*) = *p* is a proper element of *L*. Let *ab* ≤ *p* and assume that *b≰p*; that is, *b*1_*M*_ ≠ 0_*M*_. But *M* is a second *L*-lattice module; then *b*1_*M*_ = 1_*M*_. Since *b*1_*M*_ = 1_*M*_ and *ab*1_*M*_ = 0_*M*_, then *a*1_*M*_ = 0_*M*_, which implies that *a* ≤ *p*.

**Proposition 9 .**

If *M* is a secondary *L*-lattice module, then *ann*(*M*) is a primary element of *L*.

**Proof**

Suppose that *M* is a secondary *L*-lattice module. Let *ab* ≤ *ann*(*M*) and $b≰\sqrt{ann(M)}$; we prove that *a* ≤ *ann*(*M*). Since *ab* ≤ *ann*(*M*) and $b≰\sqrt{ann(M)}$, we have *ab*1_*M*_ = 0_*M*_ and (*b*)^*n*^1_*M*_ ≠ 0_*M*_ for each *n* > 0. Since *M* is secondary, we have *b*1_*M*_ = 1_*M*_. Then *ab*1_*M*_ = *a*1_*M*_ = 0_*M*_, which implies *a* ≤ *ann*(*M*).

**Proposition 10 .**

Let *L* be a *CG*-lattice. If *M* is a secondary *L*-lattice module, then $\sqrt{ann(M)}=p$ is a prime element of *L*. In this case, *M* is called *p*-secondary lattice module.

**Proof**

Let *M* be a secondary lattice. Then *ann*(*M*) is a primary element of *L* by Proposition 9. Therefore $\sqrt{ann(M)}$ is prime by [1, Lemma 2.1].

**Proposition 11 .**

Let *L* be a lattice domain and let *M* be a nonzero *L*-module. Then *M* is a second lattice module with *ann*(*M*) = 0_*L*_ if and only if *M* is a secondary lattice module with $\sqrt{ann(M)}=0_{L}$.

**Proof**

⇒: Since *M* is a second lattice module, then *M* is a secondary lattice module. Since *L* is domain, then $\sqrt{ann(M)}=\sqrt{0_{L}}=0_{L}$.

⇐: Suppose that *M* is a secondary lattice module with $\sqrt{ann(M)}=0_{L}$. Let *a* ∈ *L* and assume that *a*1_*M*_ ≠ 1_*M*_. Since *M* is a secondary lattice module, then there exists a positive integer *n* such that *a* ^*n*^1_*M*_ = 0_*M*_; that is, $a\leq \sqrt{ann(M)}=0_{L}$. Then *a*1_*M*_ = 0_*M*_. Hence, we obtain *M* is a second lattice. Clearly, *ann*(*M*) = 0_*L*_.

**Definition 12 .**

Let *M* be a nonzero *L*-lattice module. An element 0_*M*_ ≠ *N* < 1_*M*_ is said to be pure element, if *aN* = *a*1_*M*_⋀*N* for all *a* ∈ *L*.

**Proposition 13 .**

Let *L* be a *CG*-lattice, let *M* be a nonzero *L*-lattice module, and let *N* be a pure element of *M*. If *M* is a *p*-secondary lattice module, then [*N*, 1_*M*_] and [0_*M*_, *N*] are both *p*-secondary lattice modules.

**Proof**

Suppose that *M* is a *p*-secondary lattice module. Let *s* ∈ *L*. Since *M* is a secondary lattice module, then either *s*1_*M*_ = 1_*M*_ and in this case *s* · 1_[*N*,1_*M*_]_ = *s* · 1_*M*_ = *s*1_*M*_⋁*N* = 1_*M*_⋁*N* = 1_*M*_ = 1_[*N*,1_*M*_]_ or there exists a positive integer *t*, such that *s* ^*t*^1_*M*_ = 0_*M*_ and in this case *s* ^*t*^ · 1_[*N*,1_*M*_]_ = *s* ^*t*^ · 1_*M*_ = *s* ^*t*^1_*M*_⋁*N* = 0_*M*_⋁*N* = *N* = 0_[*N*,1_*M*_]_. Hence, [*N*, 1_*M*_] is a secondary lattice module.

It remains to show that $\sqrt{ann([N,1_{M}])}=p=\sqrt{ann(M)}$. Clearly, $\sqrt{ann(M)}\leq \sqrt{ann([N,1_{M}])}$. Let *r* be compact and $r\leq \sqrt{ann([N,1_{M}])}$. Since *r* is compact, there exists a positive integer *n* such that *r* ^*n*^ · 1_[*N*,1_*M*_]_ = 0_[*N*,1_*M*_]_; that is, *r* ^*n*^1_*M*_⋁*N* = *N*. Hence, we have *r* ^*n*^1_*M*_ ≤ *N*. Now we assume that $r≰\sqrt{ann(M)}$. Then *r*1_*M*_ = 1_*M*_, since *M* is secondary. Thus, 1_*M*_ = *r* ^*n*^1_*M*_ ≤ *N*; that is, *N* = 1_*M*_, which is a contradiction. Therefore, $r\leq \sqrt{ann(M)}$. Consequently, we obtain $\sqrt{ann([N,1_{M}])}\leq \sqrt{ann(M)}$.

Let *a* ∈ *L*. Since *N* is pure, *aN* = *a*1_*M*_⋀*N*. As *M* is a secondary lattice module, then either *a*1_*M*_ = 1_*M*_ or there exists a positive integer *n* such that *a* ^*n*^1_*M*_ = 0_*M*_. This implies that either *aN* = *N* or *a* ^*n*^ *N* = *a* ^*n*^1_*M*_⋀*N* = 0_*M*_. Therefore, we have *a* · 1_[0_*M*_,*N*]_ = *a* · *N* = *aN*⋁0_*M*_ = *aN* = *N* = 1_[0_*M*_,*N*]_ or *a* ^*n*^ · 1_[0_*M*_,*N*]_ = *a* ^*n*^ · *N* = *a* ^*n*^ *N*⋁0_*M*_ = *a* ^*n*^ *N* = 0_*M*_ = 0_[0_*M*_,*N*]_. Hence, [0_*M*_, *N*] is a secondary lattice module.

Now we show that $\sqrt{ann(M)}=\sqrt{ann([0_{M},N])}$. Clearly, $\sqrt{ann(M)}\leq \sqrt{ann([0_{M},N])}$. Let *c* be compact and $c\leq \sqrt{ann([0_{M},N])}$. Since *c* is compact, there exists a positive integer *k* such that *c* ^*k*^ · *N* = *c* ^*k*^ *N* = 0_[0_*M*_,*N*]_ = 0_*M*_. Since *N* is pure, we have *c* ^*k*^ *N* = *c* ^*k*^1_*M*_⋀*N* = 0_*M*_. If $c≰\sqrt{ann(M)}$, then *c*1_*M*_ = 1_*M*_. Thus, *c* ^*k*^1_*M*_ = 1_*M*_. This implies that 0_*M*_ = *c* ^*k*^ *N* = *N*⋀*c* ^*k*^1_*M*_ = *N*⋀1_*M*_ = *N*, a contradiction. Therefore, $c\leq \sqrt{ann(M)}$.

**Proposition 14 .**

Let *M* be a nonzero *L*-lattice module and let *N* be a pure element of *M*. Then *M* is a *p*-second lattice module if and only if [0_*M*_, *N*] and [*N*, 1_*M*_] are both *p*-second lattice modules.

**Proof**

⇒: Suppose that *M* is a *p*-second lattice module. Let *a* ∈ *L*. Since *N* is pure, we have *aN* = *N*⋀*a*1_*M*_. As *M* is a second lattice module, then either *a*1_*M*_ = 0_*M*_ or *a*1_*M*_ = 1_*M*_. This implies that either *aN* = 0_*M*_ or *aN* = *N*. Hence, [0_*M*_, *N*] is a second lattice module. Now, we show that *ann*(*M*) = *ann*([0_*M*_, *N*]). Clearly, *ann*(*M*) ≤ *ann*([0_*M*_, *N*]). Let *r* ≤ *ann*([0_*M*_, *N*]). Thus, we have *rN* = 0_*M*_. Now we assume that *r* *≰* *ann*(*M*). Then we obtain *r*1_*M*_ = 1_*M*_, since *M* is a second lattice module. This implies that 0_*M*_ = *rN* = *r*1_*M*_⋀*N* = 1_*M*_⋀*N* = *N*, a contradiction. Therefore, *r* ≤ *ann*(*M*).

Let *t* ∈ *L*. Since *M* is a second lattice module, either *t*1_*M*_ = 1_*M*_ and in this case *t* · 1_[*N*,1_*M*_]_ = *t* · 1_*M*_ = *t*1_*M*_⋁*N* = 1_*M*_⋁*N* = 1_*M*_ = 1_[*N*,1_*M*_]_ or *t*1_*M*_ = 0_*M*_ and in this case *t* · 1_[*N*,1_*M*_]_ = *t* · 1_*M*_ = *t*1_*M*_⋁*N* = 0_*M*_⋁*N* = *N* = 0_[*N*,1_*M*_]_. Hence, [*N*, 1_*M*_] is a second lattice module. It remains to show that *ann*(*M*) = *ann*([*N*, 1_*M*_]). Clearly *ann*(*M*) ≤ *ann*([*N*, 1_*M*_]). Let *s* ≤ *ann*([*N*, 1_*M*_]). Thus, *s* · 1_[*N*,1_*M*_]_ = 0_[*N*,1_*M*_]_; that is, *s*1_*M*_⋁*N* = *N*, and this implies that *s*1_*M*_ ≤ *N*. Now we suppose that *s* *≰* *ann*(*M*). Then, we have *s*1_*M*_ = 1_*M*_, since *M* is second. Hence, 1_*M*_ = *s*1_*M*_ ≤ *N*, a contradiction. Therefore, *s* ≤ *ann*(*M*).

⇐: Suppose that [0_*M*_, *N*] and [*N*, 1_*M*_] are both second lattice modules with *ann*([0_*M*_, *N*]) = *ann*([*N*, 1_*M*_]) = *p*. Let *r* ∈ *L*. We have two cases.

*Case 1.* If *r* ≤ *ann*([0_*M*_, *N*]) = *ann*([*N*, 1_*M*_]), then *rN* = 0_*M*_ and *r* · 1_[*N*,1_*M*_]_ = *r* · 1_*M*_ = *r*1_*M*_⋁*N* = *N*, which implies *r*1_*M*_ ≤ *N*. Thus, 0_*M*_ = *rN* = *N*⋀*r*1_*M*_ = *r*1_*M*_.

*Case 2.* If *r* *≰* *ann*([0_*M*_, *N*]) = *ann*([*N*, 1_*M*_]), then *rN* = *N* since [0_*M*_, *N*] is a second lattice module. Hence, we have *N* = *rN* = *N*⋀*r*1_*M*_, that is, *N* ≤ *r*1_*M*_, since *N* is pure. Because [*N*, 1_*M*_] is a second lattice module and *r* *≰* *ann*([*N*, 1_*M*_]); we obtain *r* · 1_[*N*,1_*M*_]_ = *r*1_*M*_⋁*N* = 1_[*N*,1_*M*_]_ = 1_*M*_. Therefore, we obtain that *r*1_*M*_ = 1_*M*_. Consequently, *M* is a second lattice module.

Now we show that *ann*(*M*) = *p*. Clearly *ann*(*M*) ≤ *ann*([*N*, 1_*M*_]). Let *s* ≤ *ann*([*N*, 1_*M*_]). Then we have *s* · 1_[*N*,1_*M*_]_ = 0_[*N*,1_*M*_]_, that is; *s* · 1_*M*_ = *N*. Thus, *s*1_*M*_⋁*N* = *N*, and so *s*1_*M*_ ≤ *N*. Now, we assume that *s* *≰* *ann*(*M*). Then, we have *s*1_*M*_ = 1_*M*_, since *M* is second. Hence, 1_*M*_ = *s*1_*M*_ ≤ *N*, a contradiction. Consequently, we have *s* ≤ *ann*(*M*).

**Definition 15 .**

An *L*-module *M* is called a multiplication lattice module if for every element *N* ∈ *M*, there exists an element *a* ∈ *L*, such that *N* = *a*1_*M*_.

**Definition 16 .**

A element *N* of an *L*-module *M* is called prime element if *N* ≠ 1_*M*_ and whenever *r* ∈ *L* and *X* ∈ *M* with *rX* ≤ *N*, then *X* ≤ *N* or *r* ≤ (*N*:_*L*_1_*M*_).

**Definition 17 .**

A element *N* of an *L*-module *M* is called semiprime element if *N* ≠ 1_*M*_ and whenever *r* ∈ *L* and *X* ∈ *M* with *r* ^2^ *X* ≤ *N*, then *rX* ≤ *N*.

**Remark 18 .**

Let *N* be a proper element of an *L*-module *M*. Then *N* is a semiprime element if and only if whenever *r* ∈ *L*, *X* ∈ *M* and *k* is a positive integer with *r* ^*k*^ *X* ≤ *N*, then *rX* ≤ *N*.

We know that a prime element is semiprime, but the converse is not true in general. The following proposition shows that the converse is true when the module is secondary and multiplication.

**Proposition 19 .**

Let *M* be a multiplication and secondary *L*-lattice module. For all element *N* of *M* such that 1_*M*_ ≠ *N* ∈ *M*, *N* is a semiprime element of *M* if and only if *N* is a prime element of *M*.

**Proof**

⇒: Suppose that *N* is a semiprime element of *M* and let *rX* ≤ *N*, where *r* ∈ *L*, *X* ∈ *M*. Since *M* is a secondary lattice module, then either *r* ^*n*^1_*M*_ = 0_*M*_ for some positive integer *n* or *r*1_*M*_ = 1_*M*_.

*Case* *1*. If *r* ^*n*^1_*M*_ = 0_*M*_, then *r* ^*n*^1_*M*_ ≤ *N*. Since *N* is a semiprime element, we have *r*1_*M*_ ≤ *N*.

*Case* *2*. If *r*1_*M*_ = 1_*M*_, then we have *X* = *rX*, since *M* is a multiplication lattice module. Then we have *rX* = *X* ≤ *N*.

Therefore, *N* is a prime element of *M*.

⇐: It is obvious.

**Definition 20 .**

Let *M* be an *L*-lattice module and let *N* be a proper element of *M*. *N* is called a primary element of *M*, if whenever *a* ∈ *L*, *X* ∈ *M* such that *aX* ≤ *N*, then *X* ≤ *N* or $a\leq \sqrt{\left(N{:}_{L}1_{M}\right)}$. Particularly, if *M* is nonzero and 0_*M*_ is primary, then *M* is said to be primary lattice module.

**Definition 21 .**

An *L*-lattice module *M* is said to be simple lattice module if *M* = {0_*M*_, 1_*M*_}.

**Proposition 22 .**

Every multiplication secondary lattice module is a primary lattice module.

**Proof**

Let *M* be a multiplication secondary module and *rX* = 0_*M*_ for some *r* ∈ *L*, *X* ∈ *M*. Now, we assume that $r\sqrt{ann(M)}$. Since *M* is a secondary module, then we have *r*1_*M*_ = 1_*M*_. Because *M* is a multiplication, then we have *rX* = *X*. Consequently, we obtain *X* = 0_*M*_.

**Proposition 23 .**

Every multiplication second lattice module is a simple lattice module.

**Proof**

Let *M* be a multiplication and second module. Since *M* is a multiplication, for every *N* ∈ *M*, there exists *a* ∈ *L* such that *N* = *a*1_*M*_. Then we obtain *a*1_*M*_ = 1_*M*_ or *a*1_*M*_ = 0_*M*_, since *M* is second. Thus, we have *N* = 1_*M*_ or *N* = 0_*M*_ for every *N* ∈ *M*; that is, *M* is simple.

**Definition 24 .**

Let *L* be a domain and let *M* be a nonzero *L*-lattice module. If *r*1_*M*_ = 1_*M*_ for every 0_*L*_ ≠ *r* ∈ *L*, then *M* is said to be divisible.

**Definition 25 .**

A nonzero *L*-lattice module *M* is said to be torsion if there exists 0_*L*_ ≠ *r* ∈ *L* such that *r*1_*M*_ = 0_*M*_.

**Proposition 26 .**

Let *L* be a domain. Let *M* be a secondary *L*-lattice module. Then either *M* is a divisible module or *M* is a torsion module.

**Proof**

Suppose that *M* is a secondary module over a domain *L*. If *M* is not divisible, then there exists 0_*L*_ ≠ *r* ∈ *L* such that *r*1_*M*_ ≠ 1_*M*_. Since *M* is a secondary lattice module, then there exists a positive integer *n* such that *r* ^*n*^1_*M*_ = 0_*M*_. Since 0_*L*_ ≠ *r* and *L* is a domain, then we have *r* ^*n*^ ≠ 0_*L*_. Consequently, there exists 0_*L*_ ≠ *r* ^*n*^ = *s* ∈ *L* such that *s*1_*M*_ = 0_*M*_. Therefore, *M* is a torsion lattice module.
